# Prognosis in Women with Breast Cancer and Private Extra Insurance Coverage

**DOI:** 10.1245/s10434-013-3025-3

**Published:** 2013-06-11

**Authors:** Juan J. Grau, Gabriel Zanon, Carlos Caso, Xavier Gonzalez, Araceli Rodriguez, Miguel Caballero, Albert Biete

**Affiliations:** 1 Department of Clinical Oncology, Faculty of Medicine, University of Barcelona, Barcelona, Spain; 2 Gynecology and Reproductive Service, Clinica Sagrada Familia, Barcelona, Spain; 3 Department of Surgery, IDIBAPS (Augusto Pi i Sunyer Memorial Institute for Biomedical Research), University of Barcelona, Barcelona, Spain

## Abstract

**Background:**

Many women covered by the Spanish public health system also have an extra private insurance policy for gynecological examinations and routine annual mammography. We retrospectively analyzed the long-term survival rates in these patients when diagnosed with breast cancer.

**Methods:**

We analyzed the survival and prognostic factors in patients diagnosed with breast cancer who were referred to a medical oncology unit for multidisciplinary treatment covered by private health insurance.

**Results:**

Between 1994 and 2009, a total of 434 patients with breast tumor were analyzed: 33 in situ and 401 infiltrating. Among the infiltrating carcinomas, 38 were stage IV and 363 were stage I, II, or III. With a median follow-up of 62 months, the 5-year global survival rate was 91 %: 97 % for stage I, 94 % for stage II, and 77 % for stage III tumors. In the patients diagnosed by routine mammography, the 5-year survival rate was 96 %, compared with 86 % in those consulting their gynecologist after breast self-examination or for other symptoms (*p* = 0.0159). Seventy-four percent were treated conservatively and experienced better survival than the 26 % who underwent mastectomy (*p* = 0.0024). Patients with disease with positive hormone receptors had a better survival rate (*p* = 0.0264); hormone receptor status was the only independent prognostic factor in the Cox multivariate analysis. Postmenopausal patients who received adjuvant tamoxifen plus exemestane had a better prognosis than those who received tamoxifen alone (*p* = 0.0203).

**Conclusions:**

Long-term survival rate was high in breast cancer patients with extra private insurance coverage. This is probably because disease was diagnosed at an early stage.

The data provided by the Spanish Oncology Society and published in the EUROCARE 4 study estimated that 19,430 new cases of cancer would have been diagnosed in Spain in 2010.^1^ The 5-year survival rate in breast cancer patients in Spain is estimated to be around 70 %, a figure similar to that found in the rest of Europe. Most of these patients are treated under the Spanish European-style public health system, which provides universal coverage for the population. The main complaints of service users are the waiting lists and the delay in carrying out diagnostic tests, such as mammography and biopsy, and surgery with curative intent.

To speed up the diagnostic process, reduce surgery waiting time, and receive better levels of comfort and service, many users of the public health system also buy extra private medical insurance. Insurance companies offer an American-style range of health services for a monthly payment. In the case of breast cancer, they offer the possibility of rapid diagnosis using image testing and biopsies and access to curative surgery generally within a week. They also offer treatments that improve patients’ psychological tolerance, such as conservative surgery or immediate breast reconstruction. However, there are no data about the association between breast cancer long-term survival rates and private extra health insurance.

We performed a retrospective study of patients with private extra health insurance referred to an oncology unit for treatment of breast tumors. We recorded patient characteristics, tumor stage at the time of diagnosis, treatment and its relation to clinical evolution, and survival in order to compare these results with those experienced by patients who receive care only via the public health system.

## Patients and Methods

### Patients

We included all consecutive patients with histological diagnosis of infiltrating or in situ carcinoma of the breast who were referred to a medical oncology specialist for multidisciplinary treatment. We followed the clinical guides for diagnosis and treatment of breast cancer of the National Comprehensive Cancer Network and the ESMO Clinical Recommendations.^2^^,^ 3 We used the annual updates and the publications of relevant studies, which have changed the treatment indications to achieve the highest level of 5-year survival rates and low morbidity and adverse effects.

### Diagnostic Tests

The diagnostic tests were defined as “at the request of the patient” when patients presented symptoms and as routine screening tests (such as annual mammography) requested by the gynecologist in women over the age of 40 years who had not presented symptoms or discovered a breast tumor by self-palpation.

At the time of diagnosis, tumor histology, hormone receptors, and other biological markers such as the *HER2* gene were recorded to establish whether they were overexpressed (positive) or not (negative).

### Treatments

Multidisciplinary treatment was considered of palliative or curative intent depending on the tumor stage at the time of diagnosis and in accordance with the recommendations of the clinical guidelines in use at the time.

Surgery in the initial stages of cancer comprised tumorectomy plus radiotherapy, except in cases of multifocal tumors or tumors more than 3 cm in diameter, in which case modified radical mastectomy (MRM) was performed. The sentinel lymph node technique was introduced in 2004 and was used when indicated.^4^ When the sentinel lymph node was invaded by tumor cells, axillary lymphadenectomy was performed. Since 1998, whenever possible, MRM has been completed with immediate or delayed breast reconstruction.

Adjuvant axillary radiotherapy was performed in patients with four or more positive lymph nodes in the axillary node clearance and in all T3 and T4.

Until 2002, adjuvant chemotherapy consisted predominantly of CMF (cyclophosphamide, methotrexate, 5-fluorouracil) for stage I disease. Since then (and in all stage II and III disease), we have used adjuvant anthracycline-based chemotherapy.^5^^,^ 6 Since 2004, cases of axillary lymph nodes with tumor invasion have also received taxanes (paclitaxel or docetaxel).^7^^–^ 9

Adjuvant hormone therapy comprised tamoxifen 20 mg/day for 5 years in premenopausal subjects and for carcinoma-in situ in the case of positive hormone receptors.^10^ In postmenopausal subjects, 2 years of tamoxifen followed by 3 years of exemestane was preferred.^11^ Since 2004, after 5 years of adjuvant tamoxifen, postmenopausal patients have received adjuvant letrozole between years 5 and 10.^12^

Since 2005, we have added adjuvant intravenous trastuzumab every 3 weeks for a year in patients with positive HER2 identified by fluorescence in situ hybridization.^13^

For neoadjuvant treatment, we used anthracycline-based chemotherapy with taxanes with or without trastuzumab, depending on biological indicators.

### Statistical Analyses

We used descriptive statistics to define patient characteristics and used parametric and nonparametric tests, such as Student’s *t* test and the Chi square test, to establish differences. For the univariate analysis of survival and disease-free interval, and response duration or progression-free survival, we used Kaplan–Meier survival curves compared with the log rank test. For the multivariate analysis of prognostic factors, we used the Cox test.

All the results were analyzed according to intention-to-treat principles.

## Results

### Patient Characteristics

Between 1994 and 2009, a total of 916 consecutive patients were referred to the medical oncology facility. Of these patients, 461 had a breast tumor. The patients had private health insurance coverage, mainly with the firms DKV Seguros (Munich Health), Asistencia Sanitaria Colegial, and Aresa-Mutua Madrileña. All patients had a histological or cytological diagnosis of infiltrating or in situ breast carcinoma requiring complementary radiotherapy with or without hormone therapy.

Of 461 patients with breast tumors, 27 consulted only to obtain a second opinion and either received treatment at other centers or were lost to follow-up. These patients were excluded from the analysis. Of the 434 remaining patients, 33 (8 %) were diagnosed with carcinoma-in situ, and those who were positive for hormone receptors were treated with tamoxifen for 5 years. The remaining 401 patients were diagnosed with invasive carcinoma and received multidisciplinary treatment in accordance with the clinical guidelines in use at the time of diagnosis. Median age was 55 years (range 29–88 years).

Hormone receptors were positive in 339 (78 %) patients, negative in 80 (18 %), and unknown in 15 (4 %). The HER2 oncogene was determined in 292 patients (67 %); all patients were treated after the year 2000, plus some who were analyzed retrospectively. HER2 was positive in 68 (23 %) and negative in the remaining 224 (77 %).

The median follow-up of living patients was 62 months. When the survival curves are broken down according to disease stage, the disease-free survival rate after 5 years was 100 % for carcinoma-in situ, 97 % for stage I disease, 94 % for stage II disease, 77 % for stage III disease, and 28 % for stage IV disease. The 5-year survival rate in these patients, including those with stage IV disease and carcinoma-in situ, was 80 %.

### Surgery

In 343 patients treated with curative intent (without metastasis), MRM was performed in 109 (32 %). MRM was performed predominantly in cases of multifocal tumors or in large tumors either because of the technical difficulty or because of the patient’s decision. The remaining 234 patients (68 %) received conservative treatment of the breast with tumorectomy or quadrantectomy with radiotherapy (or without radiotherapy in those over the age of 70 years with positive hormone receptors).

In 16 patients (5 %), a second metachronous tumor was diagnosed in addition to the synchronous bilateral neoplasms. Four of them developed a second contralateral breast cancer, which was operated and treated like another primary tumor. In the other 12 patients, cancer outside the breast was diagnosed: two in the ovary, two in the endometrium, two in the colon, two in the lung, one in the esophagus, one in the cervix, one soft tissue sarcoma, and one acute myeloblastic leukemia. All received multidisciplinary treatment with curative intent.

**Fig. 1:**
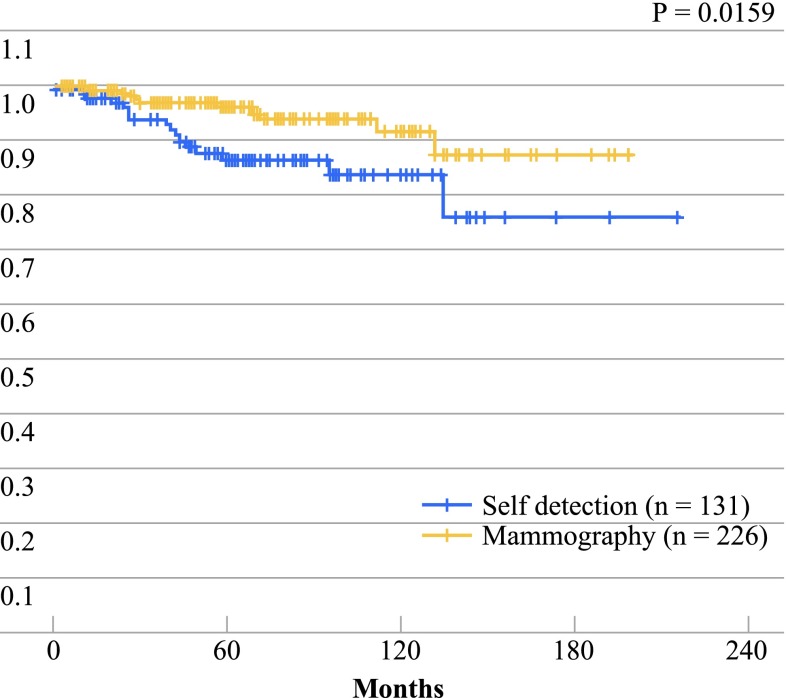
Survival according to diagnosis: self-detection or routine mammography

Three hundred fifty-seven of 434 patients (82 %) had nonmetastatic infiltrating (invasive) carcinoma and received multidisciplinary treatment. A total of 131 patients (37 %) presented a lump or tumor growth in the breast that led them to consult a gynecologist. In 226 (63 %), the diagnosis was made by the gynecologist by mammography and other imaging tests such as breast ultrasound or magnetic resonance. In this group of patients, the probability of 5-year survival was 96 % and remained above 90 % after 10 years of follow-up. Survival was significantly better than in patients who consulted their gynecologist after self-detection of a breast lump or another symptom of breast tumor (changes in the nipple, inflammation of the skin, or local pain); in these patients, the probability of 5-year survival was 86 % and above 80 % after 10 years (*p* = 0.0159) (Fig. 1).

**Fig. 2:**
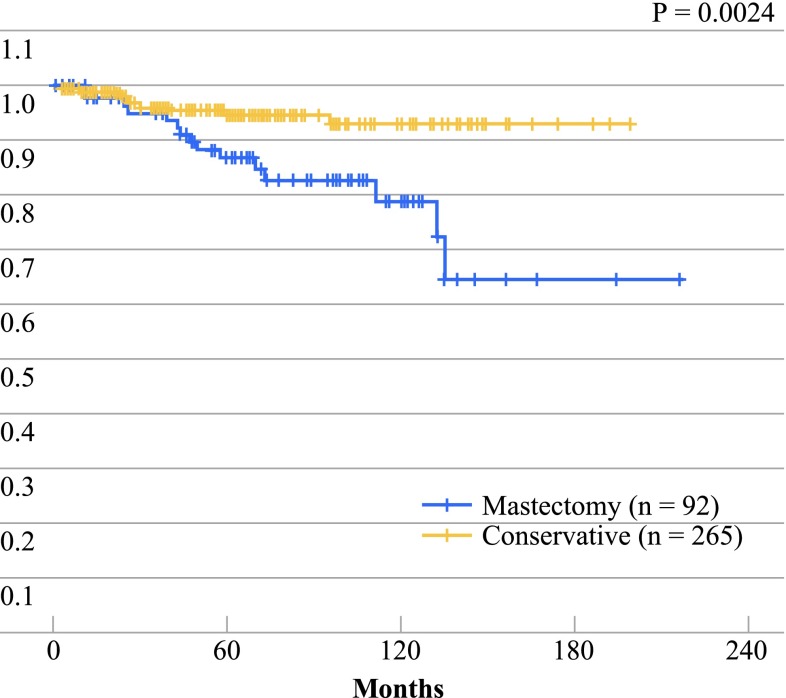
Survival of patients treated with mastectomy or conservative surgery

Patients with stage I and II disease obtained the best cure rates after 10 years—approximately 90 %, compared with less than 50 % in those diagnosed with stage III disease. In the 265 patients out of the 357 (74 %) with nonmetastatic infiltrating primary cancer who received conservative treatment, life expectancy after 10 years was above 95 % and overall survival was significantly better than in the 92 patients (26 %) who received MRM, probably because the patients treated conservatively presented earlier tumor stages at the time of diagnosis (*p* = 0.0024, Fig. 2).

### Neoadjuvant and Adjuvant Chemotherapy

**Fig. 3:**
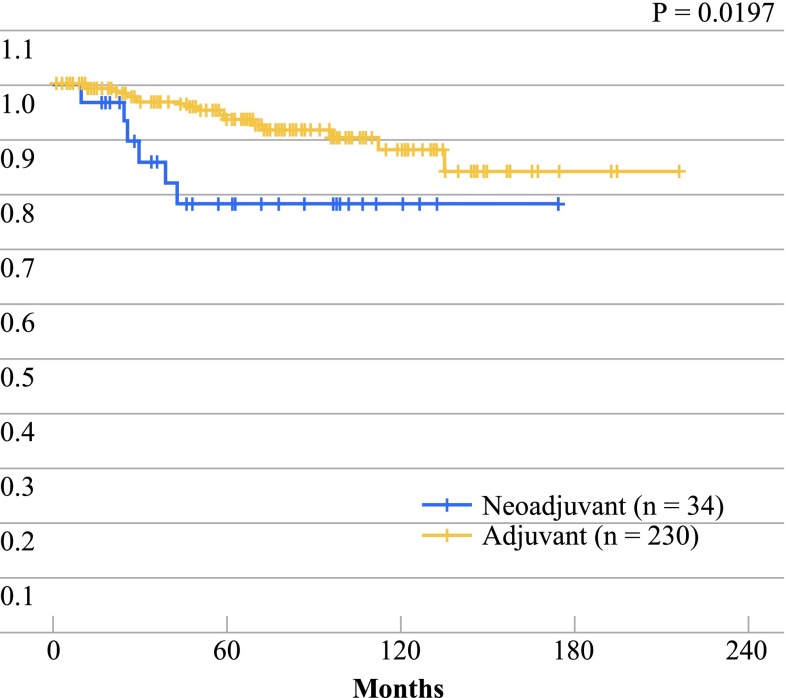
Overall survival according to treatment strategy (adjuvant or neoadjuvant chemotherapy)

In 34 of 264 (13 %) patients receiving chemotherapy for the primary tumor, induction or neoadjuvant chemotherapy was administered as the initial strategy as a result of the locally advanced state of the tumor. In all cases, chemotherapy was anthracycline based; taxanes were introduced in 1999 and in patients positive for HER2, trastuzumab in 2004. Overall survival was significantly better in the patients treated with adjuvant chemotherapy (230 patients) than in those treated with neoadjuvant chemotherapy (*p* = 0.0197), probably as a result of the earlier stage of the patients in the adjuvant treatment group. However, the cure rate, above 75 % after 5 years in patients treated with neoadjuvant chemotherapy, reflects the good prognosis achieved with this strategy of initiating treatment with chemotherapy (Fig. 3).

Since 2004, of the 230 patients administered adjuvant chemotherapy, 85 (37 %) received CMF, 101 (44 %) anthracycline-based CT, 26 (11 %) anthracyclines plus taxanes, and 18 (8 %) anthracyclines plus taxanes and trastuzumab for a year due to HER2 overexpression. The survival curves according to treatment were similar.

### Adjuvant Hormone Therapy

**Fig. 4:**
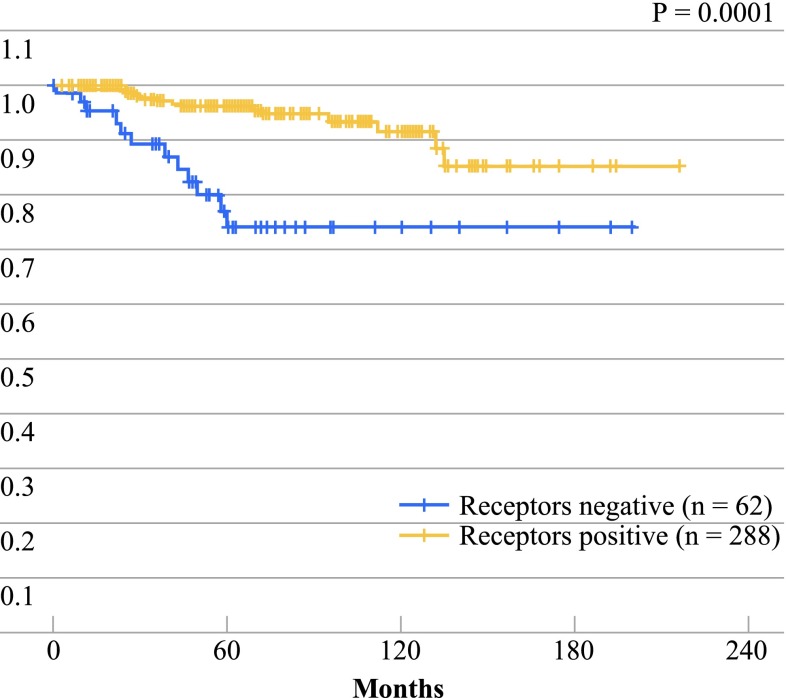
Overall survival in postmenopausal patients according to hormone receptor status

Of the 357 patients with nonmetastatic infiltrating carcinoma receiving treatment with curative intent, 288 were positive for hormone receptors and 62 were negative. In seven patients, the receptor status was unknown. At the time of the analysis, 48 % of positive receptor patients were receiving hormone therapy. Survival was significantly better in the hormone receptor-positive patients (*p* = 0.0001) (Fig. 4).

Adjuvant hormone therapy for premenopausal women with positive receptors was administered in 62 patients. Seventy postmenopausal women also received this treatment. Tamoxifen was administered to 91 patients for 2 years, followed by exemestane for three more years. A combination of tamoxifen plus letrozole was administered to 15 patients and anastrazole to 19 other patients. Hormone therapy was not administered in four patients with positive receptors as a result of intolerance or refusal on the part of the patient.

**Fig. 5:**
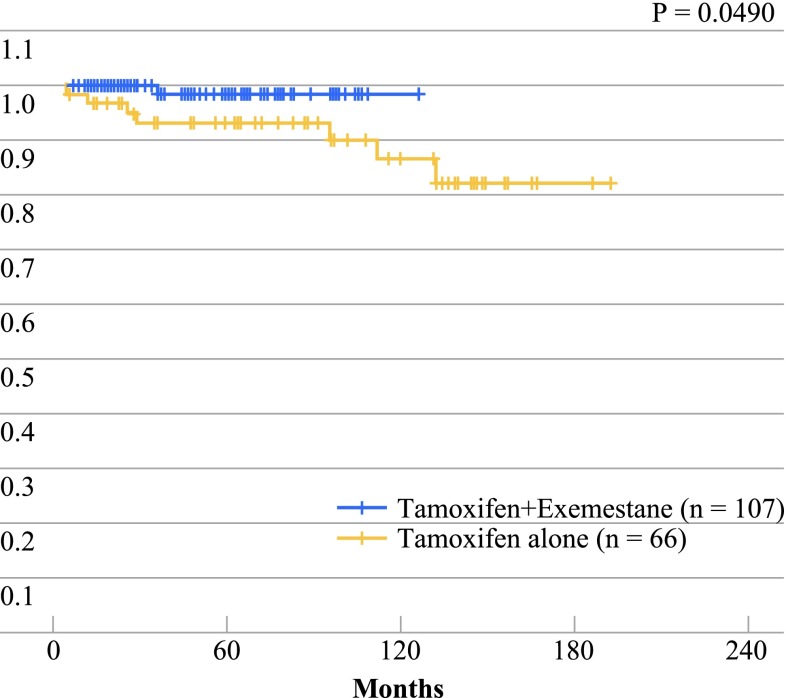
Overall survival in postmenopausal patients treated with tamoxifen or with tamoxifen plus exemestane

In the two most numerous groups of postmenopausal patients treated with adjuvant hormone therapy, overall survival was significantly better in those who received tamoxifen plus exemestane than in those treated with tamoxifen alone (*p* = 0.0490) (Fig. 5).

### HER2 Expression

Of the 254 patients in whom HER2 was determined, 200 were negative (that is, not presenting overexpression). Nine of these patients died of tumor recurrence. Fifty-four other patients were positive (that is, presenting overexpression). Six of them died from disease progression. Survival in the two groups demonstrated a trend toward statistical significance in favor of the negative patients (*p* = 0.0621), and the 5-year survival rate was 95 %. However, HER2-positive patients also obtained a high 5-year survival rate (84 %), probably as a result of the compensatory effect of trastuzumab treatment.

### Cox Multivariate Analysis

The Cox multivariate analysis included all the coded variables that had resulted in statistical significance in the univariate analysis. Of the four analyzed variables—self-examination palpation versus mammography diagnosis, stage I or II disease versus stage III disease, HER2 overexpression versus no overexpression, or positive hormone receptors versus negative—only the difference between the hormone receptors remained statistically significant (*p* = 0.0019).

### Stage IV

Thirty-nine of 434 patients (9 %) presented for the first time with a diagnosis of stage IV breast cancer. Median overall survival was 24 months. There were no significant differences in survival between patients who presented synchronous M1 at the time of diagnosis, and who therefore received treatment for M1 but also for the primary tumor (simple mastectomy, radiotherapy, etc.), and those who had been treated for primary tumor previously at another center and who later experienced recurrence (i.e., patients with metachronous M1). In the first group, 12 of 18 patients died, and in the second 18 of 20; the differences in survival in the two groups were not significant (*p* = 0.123).

### Comparison with Another Database

We compared our results with those published by EUROCARE in a similar geographic area (Granada, Spain).^14^ These patients did not have private health insurance. The 5-year survival analysis was staged according to one of five categories: small, node-negative (T1N0M0); large, node-negative (T2-3N0M0); node positive (T1-3N_M0, regardless of the number and anatomic level of the involved axillary nodes); locally advanced (T4NxM0, large tumors with skin/chest wall involvement, regardless of nodal status); and metastatic (M1) and of unspecified stage.

The 5-year survival rates by stage group in the EUROCARE study were 98, 82, 66, 63, and 46 %, respectively. In our study, they were 97, 93, 84, 78, and 21 %, respectively.

## Discussion

To our knowledge, this is the first report of the prognosis of breast cancer in patients who are entitled to receive health care through the public health system but who also have private insurance because of the subjective impression that it is more convenient and efficient. The first striking finding in this group of patients is that two-thirds were diagnosed with stage I or II disease. In a study conducted in a public health system in Granada, 37 % of patients were diagnosed with initial stage tumors, compared with 83 % in our series.^14^ In spite of the differences in the presentation of the data, the large disparity between the results suggests a clear predominance of initial stages in our study. This finding may be linked to the good prognosis of these patients, with 5-year survival rates of above 90 %.

The high rate of patients without clinical symptoms in whom breast cancer was detected during routine mammography underlines the effectiveness of the private care system, thanks to the use of annual screening for breast tumor detection and the application of standard multidisciplinary treatment.

No precise comparisons can be made with other public health systems because of the lack of statistical data, but our results suggest that the long-term survival rate for breast cancer is high in patients who take out extra private health insurance. This good prognosis may be due in part to a higher level of motivation among these patients, which leads them to request regular examinations and to comply well with treatment, but it must also be attributed to the rapid, effective response on the part of the private health system.

The use of a conservative treatment regimen is less traumatic for patients and demonstrates that the practical application of the knowledge obtained from clinical trials can offer the good results expected in a nonselected patient population.^15^ For example, patients with locally advanced cancer and a relatively poor prognosis achieve cure rates above 80 % when they receive neoadjuvant chemotherapy. Similarly, HER2-positive patients have a relatively good prognosis after the administration of adjuvant trastuzumab, and postmenopausal patients treated with tamoxifen followed by adjuvant exemestane have a better prognosis than those treated with tamoxifen alone.

In our view, the most relevant finding is the good prognosis of patients diagnosed with breast cancer by mammography before the appearance of local tumor symptoms. In this regard, the availability of early breast cancer testing offered under private insurance policies is particularly useful in places where the public system does not provide this service.

As in any retrospective study, many points are still to be resolved and analyzed, such as the psychological repercussions of effective and more conservative treatments, and the additional advantages that private health coverage can offer.
